# Perception and acceptance of a multicomponent routine program of active breaks for office workers

**DOI:** 10.47626/1679-4435-2025-1451

**Published:** 2025-09-29

**Authors:** Daniel Dias Sandy, Paula Cristina Moreira Couras Silva, Bernardo Minelli Rodrigues, Daniel Medeiros Lima, Leandra Batista

**Affiliations:** 1 Faculdade de Ciências da Educação e Saúde, Centro Universitário de Brasília, Brasília, DF, Brazil.; 2 Faculdade de Medicina, Universidade de Brasília, Brasília, DF, Brazil.; 3 Instituto Superior de Educação do Município de Itaperuna, Itaperuna, RJ, Brazil.; 4 Fisiologia e Farmacologia, Faculdade de Medicina de Campos, Campos dos Goitacazes, RJ, Brazil.

**Keywords:** health promotion, occupational health, sedentary behavior, labor gymnastics, sitting position, promoção da saúde, saúde ocupacional, comportamento sedentário, ginástica laboral, postura sentada

## Abstract

**Introduction:**

Sedentary behavior in the workplace is associated with adverse health outcomes and decreased productivity. Active breaks have emerged as a promising strategy to mitigate these negative effects.

**Objectives:**

To evaluate the effects of active breaks on well-being, productivity, and physical activity levels among 93 remote office workers (aged 18-59).

**Methods:**

Participants completed standardized questionnaires before and after the intervention, which consisted of a multicomponent program with four stages: (1) educational lectures, (2) individualized guidance, (3) support groups, and (4) the use of a mobile application. Active breaks lasted 3-5 minutes and were performed four times per day at two-hour intervals.

**Results:**

Following the intervention, 95% of participants incorporated active breaks into their daily routine (p < 0.001). Significant improvements were observed in perceived energy levels (p < 0.001), stress reduction (p < 0.001), decreased frequency of lower back pain (p < 0.001), and sleep quality (p < 0.001). Additionally, 73% reported increased vitality and reduced stress, while 88.1% expressed high satisfaction with the new routine.

**Conclusions:**

The multicomponent active break program effectively reduced the negative impacts of sedentary behavior, enhancing well-being and productivity among remote workers. The intervention demonstrated feasibility and scalability, although further research is needed to support long-term implementation in occupational health policies.

## Abstract (pt)

**Introdução:**

O comportamento sedentário no trabalho está associado a comprometimentos da saúde e da produtividade. As pausas ativas emergem como estratégia promissora para mitigar esses efeitos adversos.

**Objetivos:**

Avaliar os efeitos da implementação de pausas ativas no bem-estar, na produtividade e nos níveis de atividade física de 93 trabalhadores remotos (entre 18 e 59 anos) em regime de escritório.

**Métodos:**

Os participantes responderam um questionário antes e após a intervenção, que consistiu em um programa multicomponente com quatro etapas: (1) palestras educativas; (2) orientação individualizada; (3) grupos de apoio; e (4) utilização de um aplicativo móvel. As pausas ativas tinham duração de 3 a 5 minutos, realizadas quatro vezes ao dia, com intervalos de 2 horas entre elas.

**Resultados:**

Após a intervenção, 95% dos participantes incorporaram as pausas ativas à rotina diária (p < 0,001). Notaram-se melhorias significativas nos parâmetros de percepção: aumento da energia durante o trabalho (p < 0,001), redução dos níveis de estresse (p < 0,001), diminuição da frequência de dores lombares (p < 0,001) e melhora na qualidade do sono (p < 0,001). Adicionalmente, 73% dos participantes relataram maior vitalidade e redução do estresse, enquanto 88,1% expressaram alta satisfação com a nova rotina.

**Conclusões:**

O programa multicomponente de pausas ativas demonstrou eficácia na redução dos impactos negativos do comportamento sedentário, promovendo bem-estar e produtividade entre trabalhadores remotos. A intervenção apresenta viabilidade e potencial para aplicação em larga escala, porém vale notar necessidade de mais estudos para embasar políticas sustentáveis de saúde ocupacional.

## INTRODUCTION

The workplace — particularly in roles that involve prolonged sitting — plays a central role in managing sedentary time. Jobs that require continuous computer use can lead employees to remain seated for over 80% of the workday, increasing the risk of sedentary behavior.^1^ This behavior is associated with negative health outcomes such as fatigue, heightened stress reactivity, and musculoskeletal disorders, all of which compromise both productivity and employee well-being.^2^ Strategies such as active breaks, movement, and brief exercises have shown potential to mitigate these effects and improve quality of life in occupational settings.^1^

Moreover, prolonged sedentary time in the workplace is closely tied to excessive sitting, which has become one of the leading public health concerns globally. Sitting time is a significant risk factor for increased mortality and the prevalence of noncommunicable diseases, including cardiovascular disease, diabetes, and certain types of cancer. It is also linked to mental health issues. Beyond individual health impacts, sedentary behavior carries a substantial global economic burden by straining health care systems and affecting the financial stability of both individuals and societies.^1^ Thus, initiatives aimed at reducing sedentary behavior — especially in workplace — are critical for promoting public health and lowering the economic burden it imposes.

As society transitions from a physically active culture to a technology-driven one that minimizes physical effort, new challenges emerge regarding the sedentary habits that support seated work.^3^ Despite its adverse health effects, sedentary behavior tends to persist due to the convenience afforded by modern technology and routine-based lifestyles. Studies suggest that addressing this issue requires shared responsibility, particularly among workers themselves, to promote health and productivity. Evidence-based multicomponent interventions — such as active breaks — are recommended to reduce sitting time in workplace.^4-7^

Therefore, the present study investigated the potential effects of a multicomponent active break program implemented during the workday, with a focus on daily physical activity and workers’ self-perceived well-being and productivity. The findings may serve as a foundation for developing coordinated guidelines and actions to institutionalize active breaks as an accessible, effective strategy to mitigate the health impacts of sedentary behavior in workplace and to promote workers’ health and safety.

## METHODS

**Figure 1:**
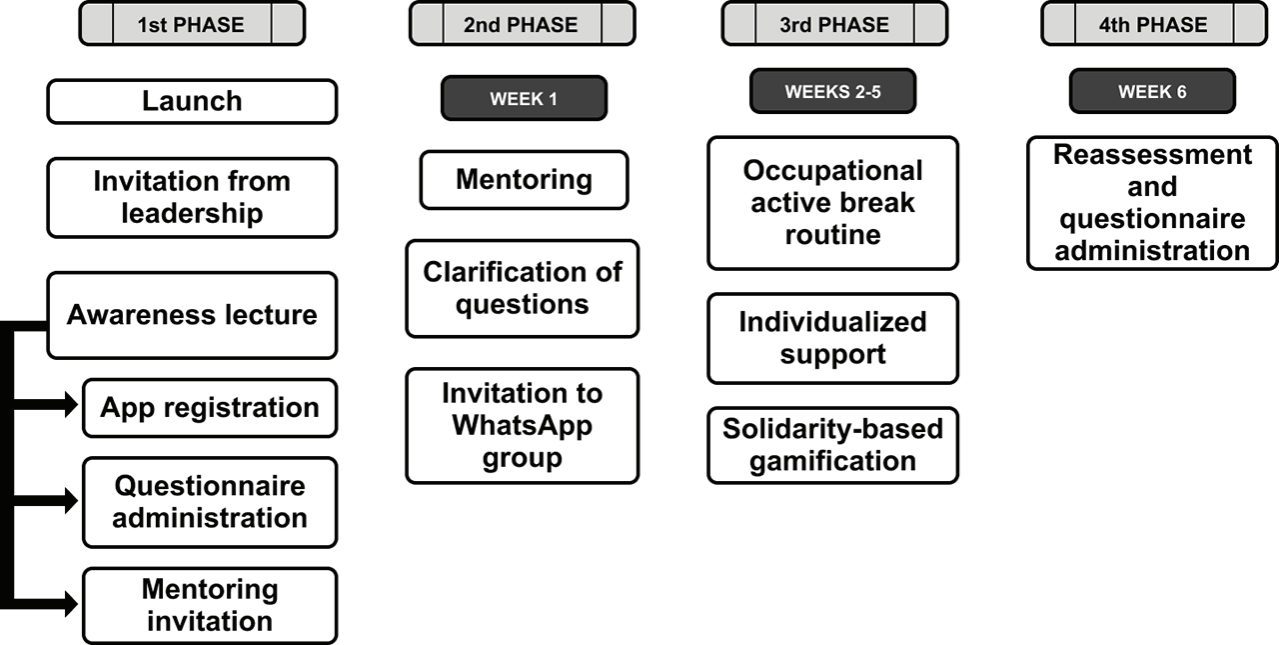
Implementation protocol procedure of the Occupational Active Break Routine^®^ program. App = application.

This was an intervention study with a quasi-experimental design, characterized as a single-arm study (without a control group) with pre- and post-intervention assessments. A multicomponent method was applied throughout 5 weeks in a real-life setting. The study included an initial lecture, an individual interview (mentoring), a support group, and the use of an application available on both desktop and mobile devices. Data collection was based on self-reports. The intervention was structured into four main phases to assess the feasibility and perceived benefits of implementing a routine of active breaks during the workday of office workers (Figure 1).

The study protocol was approved by the Human Research Ethics Committee (Protocol No. 6.335.707), and all participants were provided with an informed consent form, which was accepted remotely prior to participation in the study.

### SAMPLE

The study included 93 remote office workers of both sexes whose occupational roles and responsibilities were sedentary, working sitting at a table. Participants were eligible to participate if they met the following criteria: (1) accepted the invitation from company leadership; (2) attended the awareness lecture; (3) registered in the application (app); (4) completed the structured questionnaire; (5) agreed to participate in the study; (6) were between 18 and 59 years old; (7) were physically capable of performing activities independently; (8) were employed by the company; (9) were not on vacation during the intervention period; (10) held administrative roles performed primarily while seated at a desk with a computer; and (11) agreed to attend the mentoring session within 7 days of the awareness lecture. Interested participants were instructed to attend a presentation led by the principal investigator to receive guidance, complete registration, and undergo initial assessment.

### PROCEDURE

The first phase consisted of launching the program, which included an invitation from company leadership, followed by participation in a 1-hour remote lecture aimed at raising awareness about the risks of prolonged sitting, the importance of active breaks for productivity and well-being, and guidance on the protocol’s goals and the app’s usability. Participants were also registered through the app and given access to a scheduling channel to book a 45-minute one-on-one session for further guidance on the routine, goals, and use of the app.

Following an internal communication campaign, the 60-minute awareness and orientation lecture was scheduled remotely. All company employees were invited to register in the app, complete the initial questionnaire, download the app to their smartphones, and keep the web version open on their work computers. All procedures and activities were conducted remotely.

After the initial lecture, participants attended an individual 60-minute mentoring session, aimed at reinforcing guidance on incorporating active breaks into the daily work routine, demonstrating the app’s functionality, and promoting acceptance and understanding of its application.

Throughout 4 weeks, participants were encouraged to engage in daily active break routines. Communication and awareness-raising efforts were implemented during the first week to reinforce the study’s information and stimulate participation. All participants had ongoing access to the study researcher for questions and technical support. On the final day of the fifth week, each participant received a personalized link via a messaging app (WhatsApp^®^) to complete the follow-up questionnaire, with a deadline of up to 3 days for submission. All participants were encouraged to remain physically active and engage in recreational or sports activities during their free time. They were informed that participation in the active breaks was voluntary and incorporated into their regular working hours, in compliance with the legally mandated break periods as outlined in Article 71 of the Brazilian Consolidation of Labor Laws (CLT, 1943).

### ACTIVE BREAK ROUTINE

The active break routine consisted of four breaks throughout the workday, spaced 2 hours apart.^7^ The program was structured in the following manner: participants were first introduced to the concept of an active break, defined as a deliberate interruption of sitting time to move the body for 3-5 minutes, through activities such as jumping jacks, stretching, functional exercises, or dancing. Everyday activities such as walking to another room, hydrating, or eating were not considered active breaks.

Participants were encouraged to perform the first active break at the beginning of the workday, either by following video tutorials available in the app or individually. The second break was scheduled 2 hours after starting work, the third after returning from lunch, and the fourth 2 hours later — totaling four breaks per day, or approximately 12-20 minutes of movement. Although suggested times were based on common work schedules, participants were allowed flexibility in timing as long as the 2-hour interval between breaks was maintained.^7^

### SOCIODEMOGRAPHIC QUESTIONNAIRE

Upon registering in the app, participants completed a self-report sociodemographic questionnaire consisting of only closed-ended items. The purpose was to collect demographic data such as age, sex, height, and weight (used to calculate body mass index [BMI]), professional role (leadership or staff) as well as behavioral and emotional variables related to work. These included occupational sedentary behavior,^8,9^ frequency of daily active breaks,^10,11^ weekly exercise frequency,^12^ perceived energy and vitality at work,^13^ subjective stress perception,^14^ lower back pain,^15^ and sleep quality.^16^ Questions were derived from validated, linguistically adapted scales.

After the fifth week of intervention, the same questionnaire was administered again for post-intervention analysis, including two additional closed-ended questions to assess acceptability and participant satisfaction (Appendix 1).

### MENTORING

The individualized meeting for reinforcement and guidance on the routine and app usage was structured in three phases. In the first phase, the specialist reviewed the participant’s responses to the sociodemographic questionnaire and discussed their current situation. In the second phase, barriers, limitations, and goals for implementing the routine were identified. In the third phase, the participant described their daily work routine, and strategies were developed to incorporate active breaks at the suggested times, aiming to encourage daily practice and minimize non-compliance.^7^ All participants were informed that the active breaks did not need to be performed at exact scheduled times, but rather within the proposed time intervals.^7^

### APPLICATION

To support the implementation of active breaks, a specialized app was used to manage sitting time and promote workplace movement. Available in Portuguese and accessible on both mobile and desktop versions, the app sent scheduled reminders aligned with the guidance provided during mentoring sessions and offered guided video lessons directly on the participants’ work computers.

### DATA ANALYSIS

A qualitative and quantitative (mixed methods) approach was adopted to evaluate, compare, and assess the feasibility, acceptability, and benefits of incorporating active breaks into the workday, as well as other relevant variables.

For sociodemographic analysis, frequency analysis was used to determine participants’ age, sex, BMI, and job role for sample stratification. Descriptive statistics were then applied by frequency distribution to assess the following sample characteristics: Active Breaks (AB), Occupational Sedentary Behavior (OSB), Weekly Exercise Frequency (WEF), Perceived Energy and Vitality at Work (PEVW), Frequency Of Back Pain (FBP), Perceived Stress (PS), and Sleep Quality (SQ).

To assess program adherence, the total sample was stratified by age group, sex, and job role. Frequency analysis was then conducted pre- and post-implementation of the active break routine, both overall and by category. The Wilcoxon signed-rank test was used to compare paired samples and assess population distribution differences before and after the intervention, under the null hypothesis (H_0_) that there is no significant difference between the group distributions, versus the alternative hypothesis (H_1_) that there is a significant difference — thus assessing program feasibility.

To evaluate changes in daily movement behavior (OSB and WEF), self-perceived well-being and productivity (PEVW, FBP, PS, and SQ), and associations between these variables and the active break routine, the Wilcoxon test was again used for paired-sample comparisons to determine whether the pre- and post-intervention distributions were significantly different. The same hypotheses (H0 and H1) were applied.

Descriptive analysis was used to assess the perceived benefits and satisfaction of participants. Acceptability and satisfaction were evaluated using the number and percentage of responses per category. Open-ended responses to the question “Why?” were also analyzed to identify patterns in perceived benefits.

Statistical analyses were conducted with a significance level of p < 0.05, and p-values > 0.05 were interpreted as sufficient evidence to reject the null hypothesis in favor of the alternative. All analyses were performed using IBM SPSS Statistics for Windows, version 25.0 (IBM Corp., Armonk, N.Y., USA).

## RESULTS

Initially, 106 employees accepted the invitation to participate in the study. During the mentoring phase, 3 participants were excluded for not meeting the eligibility criteria due to dismissal, vacation, or being over the age of 60. A total of 103 participants qualified and agreed to participate in the study. Of these, 93 participants (90%) completed all study procedures — including the awareness lecture, app registration and questionnaire completion, mentoring, 4 weeks of the active break routine, and the post-intervention reassessment — and were included in data analysis.

All participants held desk-based positions involving computer use. Sample consisted predominantly of women (n = 63, 68%) and non-managerial staff (n = 67, 72%). The average age was 39 years (standard deviation [SD], 9.33 years). At the start of the study, the average BMI was 30.1 (SD, 8.34). Additionally, 29% reported not engaging in physical exercise at the time of intervention, none of the participants practiced active breaks daily, and 98% reported sitting for more than 6 hours per day on workdays (Tables 1 and 2).

**Table 1:** Frequency of active break practice before and after intervention

	Active break routine										p-value*
	Never		Rarely		Once/day		2-3 times/day		4 times/day		
	Pre n (%)	Post n (%)	Pre n (%)	Post n (%)	Pre n (%)	Post n (%)	Pre n (%)	Post n (%)	Pre n (%)	Post n (%)	
Overall											
Workers n = 93	-	-	-	**5 (5)**	-	**19 (20)**	-	**54 (58)**	-	**15 (16)**	< 0.001
Age group (years)											
18-29	-	-	-	-	-	5 (33)	-	5 (33)	-	5 (33)	< 0.001
30-39	-	-	-	3 (7)	-	8 (19)	-	25 (58)	-	7 (16)	
40-49	-	-	-	2 (7)	-	4 (15)	-	20 (74)	-	1 (4)	
50-59	-	-	-	-	-	2 (25)	-	4 (50)	-	2 (25)	
Sex											
Female	-	-	-	3 (5)	-	13 (21)	-	36 (57)	-	11 (18)	0.001
Male	-	-	-	2 (7)	-	6 (20)	-	18 (60)	-	4 (13)	
Job role					-		-				< 0.001
Leadership	-	-	-	1 (4)	-	5 (19)	-	17 (65)	-	3 (11)	
Staff	-	-		4 (6)	-	14 (21)	-	37 (55)	-	12 (18)	

n = number of respondents.

* Wilcoxon signed-rank test.

**Table 2:** Work-related behavioral and emotional indicators before and after intervention and their association with the active break routine^7^

Occupational sedentary behavior (hours per day)	Pre (n = 93)	Post (n = 93)	p-value*
	n (%)	n (%)	
More than 10	18 (19.4)	9 (9.7)	< 0.001
8-10	42 (45.2)	32 (34.4)	
5-7	31 (33.3)	37 (39.8)	
1-4	2 (2.2)	15 (16.1)	
Weekly exercise frequency			
None	27 (29.0)	10 (10.8)	< 0.001
1	11 (11.8)	5 (5.4)	
2	11 (11.8)	14 (15.1)	
3	18 (19.4)	15 (16.1)	
4	9 (9.7)	17 (18.3)	
5	9 (9.7)	18 (19.4)	
6	5 (5.4)	8 (8.6)	
7	3 (3.2)	6 (6.5)	
Energy and vitality at work			
Discouraged, no energy or vitality	11 (11.8)	-	< 0.001
Low energy and vitality	23 (24.7)	2 (2.2)	
Some energy and vitality	28 (30.1)	29 (31.2)	
Energetic and vigorous	27 (29.0)	53 (57.0)	
Highly energetic and vigorous	4 (4.3)	9 (9.7)	
Stress perception			
Constantly	5 (5.4)	1 (1.1)	< 0.001
Very often	18 (19.4)	6 (6.5)	
Occasionally	38 (40.9)	27 (29.0)	
Rarely	30 (32.3)	44 (47.3)	
Not at all	2 (2.2)	15 (16.1)	
Back pain frequency (times per week)			
Very frequently (4 or more)	7 (7.5)	3 (3.2)	< 0.001
Frequently (at least 2-3)	27 (29.0)	5 (5.4)	
Occasionally (1)	36 (38.7)	43 (46.2)	
None	23 (24.7)	42 (45.2)	
Sleep quality			
Very poor	3 (3.2)	-	< 0.001
Poor	39 (41.9)	20 (21.5)	
Good	41 (44.1)	51 (54.8)	
Very good	10 (10.8)	22 (23.7)	

n = number of respondents.

* Wilcoxon test (p < 0.05).

### ADHERENCE TO THE ACTIVE BREAK ROUTINE

At the beginning of the study, none of participants engaged in active breaks. After intervention, 95% of workers reported adopting the daily practice of active breaks over a 4-week period (p < 0.001). Of these, 5% reported rare practice, indicating low adherence to the program; 20% practiced 1 active break per day; and 54% and 16% reported implementing 2 to 3 and 4 active breaks per day, respectively, during their workday. No participant reported “Never practicing active breaks” after intervention (Table 1).

When analyzed by category, statistically significant adherence was observed across all age groups (p < 0.001), both sexes (p < 0.001), and job roles (p < 0.001) (Table 1).

### EFFECTIVENESS OF THE CONSCIOUS ACTIVE BREAK ROUTINE

Data analysis revealed statistically significant improvements (p < 0.001) in all behavioral and emotional indicators assessed after implementation of active break routine.

For OSB, a significant reduction was observed in the proportion of workers sitting for more than 10 hours per day (19.4% pre-test vs. 9.7% post-test). Simultaneously, there was a marked increase in participants who reduced their sedentary time to 1-4 hours per day, from 2.2% to 16.1%.

Regarding WEF, there was a substantial decrease in the group that did not engage in any physical activity (29% vs. 10.8%), accompanied by an increase in those who began exercising four or more times per week (19.4% vs. 37.7%).

In terms of PEVW, results showed a sharp decline in the proportion of workers feeling discouraged or with low energy (36.5% vs. 2.2%), while the category “energetic and vigorous” significantly increased (29% vs. 57%).

PS also showed notable improvement, with a drop in the frequency of high stress reports (“constantly” or “very often”) from 24.8% to 7.6%. Conversely, the proportion of participants reporting “little” or “no” stress increased from 34.5% to 63.4%.

As for FBP, there was a significant reduction in frequent reports (36.5% vs. 8.6%), while the “none” category rose from 24.7% to 45.2%.

Finally, SQ improved significantly, with a reduction in the proportion of individuals rating their sleep as “poor” or “very poor” (45.1% vs. 21.5%) and an increase in those rating it as “good” or “very good” (54.9% vs. 78.5%). These findings demonstrate that implementing active breaks is associated with significant improvements across multiple occupational behavior and self-reported well-being indicators, reinforcing its potential as a workplace health promotion strategy (Table 2).

### PERCEIVED BENEFITS AND SATISFACTION WITH THE ACTIVE BREAK ROUTINE

**Figure 2:**
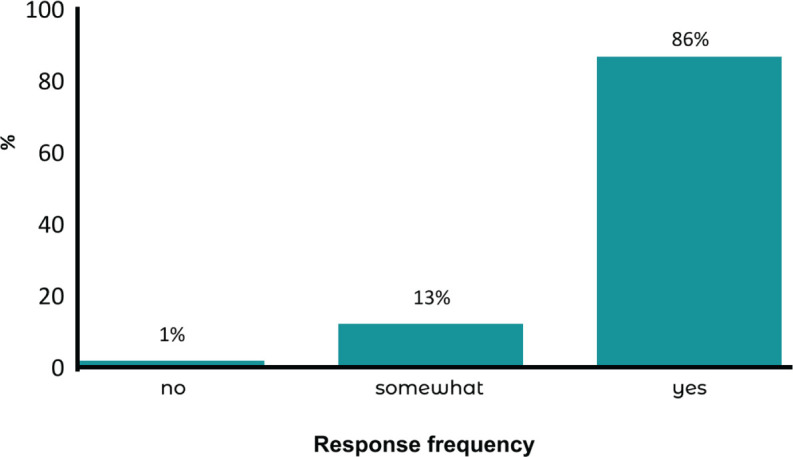
The extent to which the active break routine positively impacted participants’ productivity and emotional well-being.

In the context of implementing the active break routine, workers reported perceived benefits in both productivity and well-being, along with high levels of satisfaction with the practice (Figure 2). Open-ended responses revealed that 73% reported improvements in vitality and productivity, reductions in emotional or physical stress, reduced fatigue, and improved sleep quality. Additionally, 15% mentioned other types of benefits, and 12% reported no perceived improvement. Overall, a high level of satisfaction was observed, with a satisfaction rate of 88.1% (Appendix 1).

## DISCUSSION

The results of this study demonstrate that implementing a multicomponent active break program was feasible, well accepted, and perceived as beneficial by all participants. This finding is significant, considering the predominance of sedentary work among administrative professionals,^17^ driven by increasing automation,^18^ and cultural and organizational barriers that reinforce sedentary behavior.^17^ This context highlights the urgent need for multicomponent approaches to educate workers and mitigate health risks among those with high sedentary occupational demands.^19^

After stratifying the demographic profile of participants — since factors such as job role and age could influence adherence — the program showed high acceptance across all age groups, sexes, and job roles, with acceptance and satisfaction rates of 86% and 88%, respectively, consistent with previous studies.^20,21^ At baseline, none of participants engaged in daily active breaks, likely due to lack of awareness, habitual behavior, or organizational barriers.^17^ After intervention, daily practice increased significantly, with 95% adherence (p < 0.001), corroborating findings by Ojo et al.,^22^ who reported 91% adherence in a similar intervention using computer-based prompts.

Moreover, the frequency of taking two or more active breaks per day rose substantially to 74% (Table 1), which aligns with studies supporting the feasibility of multicomponent interventions for managing sitting time and promoting health benefits.^7,19,23^ These results indicate that the program had considerable potential to raise awareness and reinforce the importance of medium- and long-term multicomponent actions, adapted to individual needs, for sustainable adoption of healthy practices in the workplace.^24^

Several findings highlight the effectiveness of well-structured, technology-supported interventions — such as software and mobile apps that encourage reducing sitting time and promote conscious movement.^23,24^ Programs that include online self-help tools have also been shown to enhance participation in sedentary time management initiatives.^25^

Although the results are promising, the limitations of the study underscore the need for more rigorously controlled research to comprehensively assess the effects of implementing active breaks every 2 hours with technological support in real-world corporate settings. Such studies would help confirm the benefits and provide a deeper understanding of long-term impacts.

There was a consistent level of agreement and high perceived benefit among participants, consistent with Wilkerson et al.,^26^ who also reported strong acceptability of multicomponent programs involving sit-stand desks.

In open-ended responses, 73% of participants reported improvements in vitality, productivity, reduced emotional or physical stress, decreased fatigue, and better sleep quality. Meanwhile, 12% reported no perceived benefit or lack of engagement. These respondents primarily belonged to the groups who answered “Somewhat” or “No,” which confirms a predominantly positive perception overall (Appendix 1). These findings align with Rosenkranz et al.,^27^ who linked reduced sedentary behavior to improved well-being, reinforcing the potential of simple interventions, such as active breaks, to promote physical and mental health.

Although the qualitative nature and design of the study limit definitive conclusions, the results were promising — indicating benefits in sedentary behavior management and self-perceived well-being (Table 2). Additionally, there was a notable reduction in perceived sitting time, particularly among those who had been sitting for more than 10 hours per day, supporting findings from studies highlighting the effectiveness of multicomponent strategies in reducing sedentary behavior.^7^ The practice of active breaks may also contribute to increased motivation for engaging in leisure-time exercise.^5,7,26,28^

With regard to well-being, there was a significant increase in perceived energy and a reduction in stress (p < 0.001), which may be attributed to improved blood circulation, enhanced brain oxygenation, and nervous system regulation. The practice also reduced back pain and improved sleep quality (p < 0.001), possibly due to muscle activation, improved circulation, and better circadian rhythm regulation.^29^

The decision to recommend breaks every 2 hours was based on international guidelines.^5-8,11^ This frequency showed high acceptance, corroborating Rogers et al.,^29^ who found that 2-hour breaks were the most accepted option (89%). In contrast, Hargreaves et al.^30^ reported that 30-minute breaks, although beneficial, were not feasible in real-world workplace settings.

The proposed multicomponent program stood out for its accessibility, low cost, and ease of implementation, requiring no special equipment or structural changes.^30^ These features made it suitable for organizations of various sizes, reinforcing its effectiveness in promoting employee well-being and productivity. This study contributes to the growing body of evidence supporting practical and accessible technological strategies to encourage active breaks in workplace.

### STUDY LIMITATIONS

Several challenges were encountered, particularly in terms of methodological control. The main limitation was the inability to establish a control group, as the study was conducted in a real-life setting with the intervention group defined by the participating company. Secondly, all interventions and data analyses were conducted remotely, which made it difficult to control variables.

The active break routine app^7^ was used to support the practice through educational videos, visualization of the routine established during mentoring sessions, and reminders encouraging the breaks. However, due to the characteristics of the sample and the technology used, it was not possible to directly validate the performance of active breaks through the app, as there was no way to confirm whether breaks were actually taken after receiving the reminders. Additionally, many participants may have chosen to perform the breaks without using the available instructional videos, leading to the evaluation of active break frequency through self-report.

Data on sedentary behavior and sitting time were not collected using accelerometers due to logistical constraints. Instead, self-report scales were used, which may carry bias in estimating total daily sitting time and weekly physical activity.

## CONCLUSIONS

This study demonstrated that the implementation of a multicomponent program promoting active breaks was feasible as a behavioral intervention for managing sitting time in the daily routine of office workers. The high adherence and acceptability rates reinforce its potential as a promising strategy to promote well-being and productivity among professionals engaged in cognitively demanding, sedentary work.

The findings highlight the importance of practical and accessible interventions to mitigate the negative effects of sedentary behavior in occupational settings, based on participants’ perceptions of their health and well-being. However, further research is needed to confirm and expand upon these findings, as well as to strengthen strategies that support the development of inclusive and sustainable workplace health policies focused on promoting well-being.

This study contributes to the growing body of evidence supporting the implementation of active breaks as an effective approach to improving office workers perceived health and quality of life. Nonetheless, future research should directly evaluate the physiological and clinical impacts of such interventions.

## APPENDIX

**Table:**

Participant ID	Role	Sex	Has incorporating active breaks into your work routine had a positive impact on your productivity and emotional well-being?	Why?*	From 0 to 10, how likely are you to recommend the active mental routine to a friend or coworker?
1	Employee	F	Yes	*It helped me wake up, feel more energized, and motivated.*	10
2	Leadership	M	Yes	*Doing these activities consciously really helped me relax, and it also gave me moments to reflect.*	10
3	Employee	F	Yes	*Taking breaks has become part of my routine now. It was essential to complement my physical therapy sessions, and it helps me change my pace and move from a sitting position.*	10
4	Leadership	F	Yes	*I didn’t have spinal pain, felt more willing to exercise at the gym, and after the breaks I felt more energized to work.*	10
5	Leadership	F	Yes	*I felt more active and in a better mood.*	10
6	Employee	M	Yes	*With small changes in my routine, I got enough energy to do both my work and home activities. My posture and muscle pain improved. The breaks helped me focus better at work, which improved my processes and even the heavier tasks.*	10
7	Employee	M	Yes	*They help us disconnect from work and reconnect with ourselves, which relieves daily stress.*	10
8	Employee	F	Yes	*Breaks during the day recharge me and give me fresh energy.*	10
9	Employee	M	Yes	*I feel more energetic during work hours, especially after lunch.*	10
10	Employee	F	Yes	*Starting the day with some exercises makes me feel more ready for the day’s activities.*	10
11	Employee	F	Yes	*At the end of my first pregnancy, I felt much more tired. Even though I didn’t manage to do the breaks and exercises the way I wanted this time, I already feel a big difference — I’m much more active now.*	10
12	Employee	F	Yes	*It improved my concentration, energy, and mood.*	10
13	Employee	M	Yes	*I feel more emotionally prepared to deal with work challenges, and my self-**esteem has improved.*	10
14	Employee	F	Somewhat	*I couldn’t really fit the breaks into my routine. Instead, I exercised after work twice a week.*	8
15	Leadership	F	Yes	*It encouraged me to take breaks and gave me mental rest.*	10
16	Leadership	M	Yes	*It mainly helped me at bedtime — I started doing music therapy.*	10
17	Leadership	M	Yes	*The breaks helped me a lot. Daniel’s guidance opened my eyes to things I hadn’t noticed before. I started walking my son to school, taking the stairs for exercise, changed my eating habits, and even lost some weight. Thank you so much for everything.*	10
18	Employee	F	Yes	*Because it makes us stop consciously.*	10
19	Employee	M	Somewhat	*Unfortunately, I couldn’t dedicate myself as much as I wanted because of work overload. But I believe a lot in the program and think it would be great if we could expand it.*	7
20	Employee	F	Yes	*It reminded me to stop at times when I normally wouldn’t.*	10
21	Employee	F	Yes	*It was a way to formalize something I was already doing.*	10
22	Leadership	M	Yes	*I felt more clear-headed and less tired.*	10
23	Leadership	M	Yes	*Yes, it really opened my eyes!*	10
24	Employee	F	Yes	*I noticed I was more productive during work hours.*	10
25	Leadership	F	Yes	*I used to exercise before work, but I didn’t realize how important it was to take breaks during the day. I’d sit for 4 hours or more straight at the computer. With the program’s encouragement, I got the courage to start biking to and from work. I even agreed with Daniel that when I use the car, I’ll take the stairs — all 11 floors!*	10
26	Employee	M	Yes	*I think it helped me build healthier habits. I started using “useless” parts of my routine to recharge energy and motivation. I even cut back on coffee — now I don’t feel like I need it anymore!*	8
27	Employee	F	Yes	*I noticed that on days I didn’t take breaks, I felt tired, unmotivated, and less productive.*	9
28	Employee	F	Somewhat	*Too much work and stress kept me from taking breaks the way I should.*	6
29	Leadership	F	Yes	*It became a daily habit. It pushed me out of my comfort zone and made me more active, especially in the mornings.*	10
30	Employee	F	Yes	*It makes me feel energized and upbeat.*	9
31	Leadership	F	Yes	*I sleep better now and pay more attention to my body’s needs.*	10
32	Employee	F	Yes	*After doing the routine, I always felt more focused and energized. Even though I know I need medical help, at least I became aware that every day I can try to make it a better one.*	10
33	Leadership	F	Yes	*I managed to make time for both my body and mind.*	10
34	Employee	F	Yes	*The active breaks were great for boosting my work.*	10
35	Employee	M	Somewhat	*Even the little I managed to do helped my well-being.*	7
36	Employee	M	Yes	*The breaks and exercises made me more engaged in my daily activities.*	9
37	Leadership	M	Yes	*I already feel much better — more active and motivated.*	10
38	Leadership	F	Yes	*Changing my habits and practicing daily exercises, small breaks, and meditation made all the difference in my physical and mental health!*	10
39	Employee	F	Yes	*I felt more energetic and active in the afternoons.*	9
40	Employee	M	Yes	*Even though the Active Mental Routine happened during some very stressful weeks at work, the program really improved my productivity and, above all, my emotional well-being.*	10
41	Leadership	F	Yes	*Definitely. It makes work lighter for both body and mind, leaving you more willing to do daily tasks.*	10
42	Employee	F	Yes	*I see the activities as a kind of restart that helps me get back on track. Moving the body doesn’t just wake it up — it also refreshes the mind.*	10
43	Leadership	F	Yes	*I felt more energetic and alert, especially in the mornings when I usually had low energy.*	9
44	Employee	F	Yes	*I realized how important these breaks are. They gave me more energy and left me feeling much more ready to go.*	10
45	Employee	F	Yes	*My stress, tiredness, and daily sleepiness decreased. My productivity went up, and my personal life improved a lot too. Now I have more energy for housework and leisure. Before, everything felt overwhelming — like I had no time and was about to snap. With Daniel’s help, I managed to organize my routine and see the positive side of things.*	10
46	Employee	F	Yes	*It helped relax both my body and mind.*	10
47	Employee	F	No	*Unfortunately, I couldn’t start the program because of personal reasons and the phase I’m going through.*	10
48	Employee	F	Yes	*I felt much better, more energetic, and refreshed.*	10
49	Leadership	F	Yes	*I’ve been going to the office more often, which made me include breaks more regularly. As a result, I’ve been feeling less stressed.*	9
50	Employee	F	Yes	*It boosted my energy.*	10
51	Employee	F	Yes	*It made me more alert.*	10
52	Employee	F	Yes	*With the active routine, I noticed my sleep improved — I wake up energized every day, I exercise more, and I perform better overall.*	10
53	Leadership	M	Yes	*Sometimes you just need to switch off a bit, breathe, and stretch — and I wasn’t doing that. These small breaks had a big impact over the weeks.*	10
54	Employee	F	Yes	*I learned the importance of taking breaks throughout the day and the positive effect they bring.*	10
55	Employee	F	Yes	*I had more energy during the day, especially in the evenings when I would normally feel exhausted.*	10
56	Employee	F	Yes	*I had less back pain and noticed a boost in productivity.*	10
57	Employee	F	Yes	*I feel more energetic at the end of the day.*	10
58	Leadership	F	Yes	*Even though I couldn’t do all the breaks, it made me aware of their importance. I made an effort to prioritize self-care without replacing what I needed to do for myself, even when work was urgent. For example, I managed to stick with morning activation and made sure to get up and stretch.*	9
59	Employee	F	Somewhat	*I couldn’t practice very often.*	8
60	Employee	F	Yes	*I feel more energetic.*	10
61	Employee	M	Somewhat	*I still haven’t managed to fully adjust my routine to follow the plan. It would be great if the app had alarms for the scheduled tasks.*	9
62	Employee	F	Yes	*It helps me relieve stress and focus more on my work.*	8
63	Employee	F	Yes	*Every time I do the exercises, I feel more energized to keep going. The pain I used to feel has gone down a lot.*	10
64	Employee	F	Yes	*Taking breaks during the day makes you come back recharged and with a clearer mind.*	10
65	Employee	F	Somewhat	*Because I don’t do it often.*	10
66	Employee	M	Yes	*It helps me get ready for the day, relieve stress, and build more awareness.*	10
67	Employee	F	Somewhat	*You feel more energized.*	8
68	Employee	F	Yes	*I feel more energetic and lively.*	10
69	Leadership	F	Yes	*It makes you more aware of the need to move.*	10
70	Leadership	M	Yes	*It breaks the routine and makes you come back more motivated and alert.*	10
71	Employee	F	Yes	*Since I don’t like going to the gym, I started adding walks and small movements — like jumping jacks or squats — during the day. That way it doesn’t feel like “exercise,” just a light and easy routine.*	10
72	Employee	M	Yes	*I felt more energized when I followed the routine.*	10
73	Leadership	M	Yes	*I feel more willing and energized.*	9
74	Leadership	F	Yes	*I feel so much better — more energetic, happier with my body, sleeping better, in a better mood, everything.*	10
75	Employee	F	Yes	*I don’t feel pain anymore (especially headaches). I have energy after lunch, I fall asleep much faster and earlier — before I used to go to bed after 11 p.m., now by 9 p.m. I’m already really sleepy.*	10
76	Employee	M	Yes	*I have more energy and better mobility.*	10
77	Employee	M	Yes	*It helped me change my routine.*	10
78	Employee	M	Yes	*It made me reflect on my posture and helped me get into the habit of taking conscious breaks, which gave me better awareness.*	10
79	Leadership	M	Yes	*It reduced my back pain and gave me more energy throughout the day.*	10
80	Leadership	M	Somewhat	*There was improvement, but it still needs work.*	10
81	Employee	M	Yes	*I felt more energized, especially in the mornings when it usually takes me longer to “wake up.” Plus, the active walks turned into a chance to interact with others doing them too.*	10
82	Employee	F	Yes	*Besides reactivating my body, it gave me more energy and helped reduce work tension.*	10
83	Employee	F	Somewhat	*It reduced my pain and gave me more energy.*	9
84	Leadership	M	Yes	*It keeps me active.*	10
85	Employee	F	Yes	*I feel more motivated.*	10
86	Employee	F	Yes	*I feel more energized after work.*	8
87	Employee	F	Yes	*I’m more energetic, no longer feel that extreme fatigue, and can climb stairs without losing my breath. It’s helped both my back and my anxiety. I went back to training and eating well, I’m losing weight, and I feel more confident. And it all started with the active break.*	10
88	Employee	F	Yes	*It showed me that every movement counts. Even small things can make a positive impact. My answers weren’t as positive in the last two weeks because I had COVID and am still recovering. I kept up the activations but had to pause physical activities. Still, I replaced parts of my bus commute with walking — sometimes even longer walks.*	10
89	Employee	F	Yes	*I feel more energized and less tired.*	10
90	Employee	M	Yes	*On the mornings I managed to do it, I felt more upbeat and willing.*	10
91	Employee	F	Somewhat	*Yes, but since it also depends on me, it can be tricky. Having mentoring, though, gives me extra motivation.*	10
92	Employee	M	Somewhat	*I don’t know.*	8
93	Employee	F	Yes	*I felt way more energized, more active, and more alive. It really made a difference in my day-to-day life.*	10

F = female; M = male.

* The responses presented in the “Why?’” column have been translated based on the faithful transcription of the participants’ records, without editing of content or form, so as to preserve the authenticity of the data.
